# Parent Perception of School Meals in the San Joaquin Valley during COVID-19: A Photovoice Project

**DOI:** 10.3390/nu15051087

**Published:** 2023-02-22

**Authors:** Tatum M. Sohlberg, Emma C. Higuchi, Valeria M. Ordonez, Gabriela V. Escobar, Ashley De La Rosa, Genoveva Islas, Cecilia Castro, Kenneth Hecht, Christina E. Hecht, Janine S. Bruce, Anisha I. Patel

**Affiliations:** 1 Department of Pediatrics, Stanford University School of Medicine, 3145 Porter Drive, Office F110, Stanford, CA 94304, USA; 2 Dolores Huerta Foundation, Bakersfield, CA 93303, USA; 3 Cultiva La Salud, Fresno, CA 93703, USA; 4 Nutrition Policy Institute, Agriculture and Natural Resources, University of California, 1111 Franklin Street, 5th Floor, Oakland, CA 94607, USA

**Keywords:** food insecurity, school meals, photovoice

## Abstract

School-based nutrition programs are crucial to reducing food insecurity. The COVID-19 pandemic adversely impacted students’ school meal participation. This study seeks to understand parent views of school meals during COVID-19 to inform efforts to improve participation in school meal programs. Photovoice methodology was used to explore parental perception of school meals in San Joaquin Valley, California, a region of predominately Latino farmworker communities. Parents in seven school districts photographed school meals for a one-week period during the pandemic and then participated in focus group discussions and small group interviews. Focus group discussions and small group interviews were transcribed, and data were analyzed using a team-based, theme-analysis approach. Three primary domains emerged: benefits of school meal distribution, meal quality and appeal, and perceived healthfulness. Parents perceived school meals as beneficial to addressing food insecurity. However, they noted that meals were unappealing, high in added sugar, and unhealthy, which led to discarded meals and decreased participation in the school meal program. The transition to grab-and-go style meals was an effective strategy for providing food to families during pandemic school closures, and school meals remain an important resource for families experiencing food insecurity. However, negative parental perceptions of the appeal and nutritional content of school meals may have decreased school meal participation and increased food waste that could persist beyond the pandemic.

## 1. Introduction

Food insecurity (FI), defined as reduced access to affordable and nutritious food, had been decreasing in U.S. households with 14.9% of households experiencing FI in 2011 to 10.5% reporting FI in 2020 [1]. Overall, during 2020, the first year of the COVID-19 pandemic, prevalence of FI was 14.8% among U.S. households with children [1]. Due to the social and economic disruption from the pandemic, by some estimates FI in households with children climbed upwards of 27.5% at one point during the pandemic [2,3]. FI has many adverse consequences for children’s physical and mental health, with increased risk for obesity [4], diabetes [5,6], and depression [7]. It has also been shown to be associated with poor maternal health, increased parental depressive symptoms, and increased conflict between parents [8], all of which affect the social and emotional development of children. FI disproportionately affects families of color [1], and thus addressing childhood FI is crucial to achieving health equity.

The National School Lunch Program was established in 1946 to address food insecurity and continues to do so, serving ~30 million US children daily. The 2010 Healthy, Hunger-Free Kids Act aligned nutrition standards for school meals with the Dietary Guidelines for Americans, increasing whole grains, eliminating trans fats, establishing appropriate calories by age, increasing required servings of fruits and vegetables, and reducing sodium. Thus, school meal programs not only reduce FI, but also promote higher overall diet quality, may decrease obesity and related health consequences, and have the potential to reduce learning losses stemming from the COVID-19 pandemic [9,10,11].

Despite the numerous benefits of school meals, there was a decrease in participation in school meal programs early in the pandemic [12]. During the COVID-19 pandemic when schools closed and switched to remote learning, the USDA granted waivers that allowed for flexibilities both in the logistics of distributing school meals as well as existing nutritional standards so schools could more easily distribute food to children given local COVID-19 safety protocols and remote schooling during the pandemic [13,14,15]. School districts developed creative solutions in their distribution of school meals for consumption at home, some delivering meals via school buses while others provided “grab-and-go” meals which parents picked up at designated schools [16,17]. 

For many parents, this was the first glimpse of what their children eat at school. Parental perceptions of school meals affect whether students participate in school meal programs [18]. Conducting this study during the period of the pandemic when schools were closed for in-person instruction, and meals were consumed at home allowed unprecedented insight into parent experience of school meals, which could persist beyond the pandemic period. 

This study was conducted in California’s San Joaquin Valley (SJV), which is a predominately rural area home to a majority Latino population including many agricultural workers [19] among the hardest hit economically by the COVID pandemic [20,21,22]. Rural areas produce most of the nation’s food, yet 9 of 10 counties with the highest rates of food insecurity are rural. Families who are of Latino background are also most affected; 1 in 6 Latinos experience food insecurity, a rate 2.5 times that of Whites [23,24]. One study of accessibility to produce in the SJV found that while most participants reported physical access to produce was not a problem in their communities, 65% reported concerns about affordability of fruits and vegetables in grocery stores and other retail locations [25]. The pandemic only further limited access to affordable healthy produce, making school meal programs an especially important source of nutrition for children in SJV [26]. 

The goal of this study was to explore parent perspectives of the school meal programs and to empower parent participants to use study findings to influence local school meal programs and enhance participation in the program. To our knowledge, the current study, which stems from concerns about meal quality and appeal mentioned in earlier parent focus group discussions conducted by our community-academic partnership [17], is one of the first U.S. studies to examine parents’ concerns regarding school meals provided during the COVID-19 pandemic and thus offers important insight that may inform efforts to address decreased school meal participation [27]. Moreover, this study provides perspectives of Latino families from rural communities who face unique challenges that are rarely explored. 

## 2. Materials and Methods

### 2.1. Community-Academic-Policy Partnership 

This study was conceptualized and conducted by a community-academic-policy partnership. The partnerships included two community-based non-profit organizations, Dolores Huerta Foundation and Cultiva la Salud, that work to advance health equity and social justice for predominately Latino farmworker communities in SJV, academic research partners at Stanford University, and policy partners at the University of California’s Nutrition Policy Institute (NPI). The study was conducted in the SJV where the two community partners are based. 

Community and policy partners collaborated with the academic partners on the entire research study. They identified the study objectives, recruited participants, and helped shape focus group discussion guides. All community, academic, and policy partners met regularly to inform the study design, data collection, and analysis processes and to review updates and provide feedback. 

### 2.2. Study Design 

Photovoice is a community-based participatory research method through which photographs are used to promote dialogue and give voice to community members’ lived experiences [28]. This methodology was selected for the current study as it was identified as an effective way to engage meaningfully with community members and to amplify their voices to advocate for changes in practices and policies. This study received approval from the Stanford University IRB. 

### 2.3. Data Collection

Participants were recruited by community partners. Inclusion criteria included (1) being a parent or caregiver of a child enrolled in a public elementary, middle, or high school in SJV, (2) speaking either English or Spanish, and (3) participating in the school meals program during the COVID-19 related school closures. The convenience sample consisted of parents of children enrolled in eight school districts across the SJV region. Parents attended a training webinar (Spanish/English) hosted via Zoom by the research team to receive instructions for photographing school meals and to review the project. Interested parents then signed up to take photographs and participate in the subsequent focus group discussions or interviews. Parents were asked to take photographs of all food provided by the school during the week of 11/2/20 to 11/6/20 and were instructed to have any food labels with nutrition information visible in the photographs. Parents were provided with cards to include in the photos on which they filled out the date, number of meals displayed in photographs, type of meals (breakfast, lunch, or snack), school where they picked up the meal, and any other comments for the photos. Photos taken by participants were sent to the study team by email or text message and then uploaded to a centralized database for the research team to organize and label. Thirty-seven participants from eight school districts provided photographs.

Six focus group discussions and two small group interviews [29] were then conducted virtually over video conference. Each discussion was led by a member of the Stanford research team with a community partner representative present in each group. Each focus group and small group interview began with the virtual sharing of one photograph selected by each parent and the open-ended question, “Please tell us about the photo you selected and what it means to you.” The facilitator then moved on to questions about school meals during the pandemic following a focus group guide, which was developed in collaboration with community partners and was informed by prior discussions with community members about their experiences with school meals. Each focus group and small group interview consisted of parents from one school district and was conducted in English or Spanish. The groups were divided in this manner to accommodate language preference and encourage discussion among parents in the same districts. This allowed for in-depth conversation about parent experiences [30] within a district for eventual action to influence policy and practice, which is a key component of photovoice methodology [28,31]. The small and large group discussions achieved thematic saturation, so no additional groups were conducted. Themes addressed in the discussions included child opinions of meals, parent opinions about quality of food, food waste, packaging and presentation of meals, and ideas for improvements. All participants received a $50 gift card. 

### 2.4. Analysis

Focus group discussions and small group interviews were audio-recorded and transcribed. Transcripts in Spanish were translated into English. All transcripts were uploaded into Dedoose, a qualitative software used to share transcripts, code data, and test interrater reliability [32]. Thematic analysis was conducted using an iterative approach. Initially, two coders developed a draft codebook, which was edited with input from team members and community partners. The two researchers then independently coded the transcripts using the code book. Discussions regarding discrepancies in coding were held until a final code book was agreed upon. The pooled Cohen’s κ score on the final code book was 0.89. 

## 3. Results

A total of 27 parents, all female, from seven school districts in the SJV participated in six focus group discussions and two small group interviews to converse about their photographs and school meals during the pandemic. Five group discussions were conducted in Spanish, and one was in English. One small group interview was in Spanish, and one was in English. Focus group size ranged from four to seven participants. The small group interviews had two to three participants each. Participants were parents of elementary, middle, and high school children with ages ranging from 5 to 17 years old. 

Three primary themes emerged from analysis of the discussions: (1) benefits of meal distribution during school closure, (2) meal quality and appeal, and (3) perceived healthfulness of meals. Representative quotes and photographs for each theme are presented in Table 1 (Theme 1), Table 2 (Theme 2), and Table 3 (Theme 3). 

Theme 1: Parent Descriptions of the Benefits of School Meal Distribution during COVID-19 Pandemic School Closures

Parents described several benefits of the transition to grab-and-go meal distribution including schools’ efforts to ensure that the logistics of picking up meals were streamlined, financial impact of schools providing meals for the family, and COVID-19 safety protocols enforced by schools providing meals. Parents appreciated that they could pick up meals at any nearby local school. Many parents mentioned the convenience of being able to pick up batched meals, meaning they could pick up several days’ meals at the same time, which meant fewer trips to the pick-up sites. They also valued efforts by schools to communicate about their meal distribution through multiple platforms including phone, email, banners, and flyers. 

Parents reported that the school meals saved them money on groceries during the pandemic, and they specifically valued receiving bulk items such as cartons of milk, dried beans, and rice (Table 1b). Parents appreciated the reliable supply of milk that they received with batched meals as they perceived it as a healthy, yet costly, food staple. One parent even stated that the milk was the main reason they continued to pick up meals for their family. Parents described bulk dried items, primarily beans and rice, such as those in Table 1b(ii), as enabling them to cook culturally preferred foods at home. One parent noted that she was experiencing unemployment during the pandemic and that she felt relief that the school meal program provided her children with consistent access to food. 

Parents also reported feeling safe collecting meals from the school sites during the pandemic. The photo in Table 1c shows meals picked up in plastic bags, which parents equated to schools’ strong safety and cleanliness standards when packaging and distributing foods. 

Theme 2: Parents Perceptions of the Quality and Appeal of School Meals during the COVID-19 Pandemic

Issues of meal quality and appeal were prominent during the photovoice discussion sessions. Parents reported throwing away meals because their children refused to eat the food. Even when children were hungry, parents said they declined to eat much of the food provided by school because it did not look appetizing or appealing. Parents described food as soggy, bruised, squished, greasy, or frozen. The photo in Table 2d depicts a pizza roll that was squished by the time it made it home. Pizza was one of the most common food items reported to be discarded and was described as greasy, rubbery, and tasteless. Both photos in row a of Table 2 demonstrate slices of pizza that were unappealing for different reasons. Photo (i) reveals grease pooled beneath the slice while photo (ii) shows a shrink-wrapped slice that is squished and misshapen. Several parents also described produce as inedible due to being bruised, discolored, or even moldy. Many parents reported that children did not like the taste of the food, and several parents described tasting the food themselves and agreeing with their children. Parents consistently reported guilt about the amount of food thrown away. Multiple parents stopped picking up meals because of the waste. 

Other concerns about school meals included lack of variety in foods. Parents perceived that repetitive meals each week made their children less likely to eat the food. Parents described putting in additional preparation to improve variety and appeal of meals. Food often required defrosting, cooking, or adding extra ingredients. Parents repeatedly expressed concern about safety of children needing to microwave food, especially with some foods being packaged in non-microwave safe packaging. The sandwich pictured in Table 2c has frozen filling that required disassembling the sandwich to defrost. These added steps to prepare meals were barriers to student consumption of school-provided meals. 

Theme 3: Parents’ Perceptions of the Healthfulness of School Meals Provided during the COVID-19 Pandemic

Nearly every parent expressed concern about unhealthy food provided in school meals. Parents believed the food was “too sweet” and unhealthy. Table 3b(i) shows waffles and pop-tarts provided for breakfast that were perceived as too sugary. Pop-tarts and breakfast cereals were common items about which parents expressed concern. Parents also noted that many of the snack foods received as a part of lunch were unhealthy (e.g., Cheetos, chips, cookies). Several parents went as far as to describe the food their child received as “junk”. A few parents also described the cheese in several foods, specifically in macaroni and cheese, cheese sandwiches, and pizza, as greasy and fatty. 

Parents reported that their children preferred the fresher, healthier components of the meals such as whole fruits and vegetables. Children reportedly preferred whole produce over packaged sliced produce; they often described the pre-sliced and prepackaged vegetables as slimy. The photo in Table 3a contrasts sliced packaged cucumbers and carrots that children perceive as slimy or spoiled next to whole apples that were more appealing. Many school districts did provide whole apples and oranges; however, these did not make up the majority of fruit and vegetable servings. 

Overall, parents expressed concern that school meals set a poor example of a healthy lifestyle for children. Parents felt that the school was missing an opportunity to teach children healthy habits through the school meals program. Several parents expressed concern that children were not getting appropriate nutrients, and those unhealthy meals would affect children’s ability to learn in school. Multiple parents also worried that children would not develop healthy habits since they lacked access to nutritious food at school. Parents were hopeful about bringing change to the food provided by their school districts. One parent stated that the district leadership is likely unaware of the poor quality and nutritional content of food being sent home, and parents expressed that they felt photographs from this study would be effective to inform school district leadership of their concerns, as was intended from the outset of this photovoice project.

## 4. Discussion

Grab n’ go school meals provided during pandemic-related school closures provided a rare opportunity for parents to see the types of foods and beverages provided in school meals. We found that parent perceptions of quality, appeal, and healthfulness of meals may have contributed to the lower participation in school meals observed during the pandemic. Our findings are particularly salient given that effective this school year, California provides all public-school children with school breakfast and lunch daily, at no charge, regardless of family income [33]. While it is important to acknowledge that school meals may have changed during the pandemic due to supply chain disruptions and USDA waivers for school meal nutrition standards, parents’ perspectives of school meals during the pandemic could persist even after school meals return to normal, thereby limiting the participation in California’s universal school meals program unless action is taken to address parent concerns. 

In our study, parents perceived meals provided during the pandemic as unhealthy, and specifically voiced that meals were too sugary. Parents in one other U.S. study with 101 parents of children (78.2% were non-Hispanic Black or Latino/Hispanic) receiving meals from New York City public schools during the pandemic expressed some similar concerns to those raised in our SJV focus group discussions about healthfulness of meals, noting foods that were more snacks than meals and often included ultraprocessed items [27]. However, in contrast to our study, parents in this New York City sample identified Nutritional Quality as a positive facilitator of participation in school meals [27]. Another study of parent perspectives of school meals conducted in a majority Non-Hispanic Black population (51.6%) found that, after the implementation of HHFKA, nearly 20% of parents perceived school meals as unhealthy while 80% viewed the meals as healthy [34]. The study populations and locations are very different among these two studies and our study, with the two existing studies examining urban populations, which may contribute to differences in food provided or affect parental perceptions of food provided by school meal programs. 

Federal nutrition guidelines for school meals, according to the HHFKA, include limitations on overall calories, sodium, added trans fats, and saturated fats. They also require meals to be whole grain-rich, offer one cup of low fat or fat-free milk, and include fruit and vegetable servings. Despite the fact that HHFKA requires meal program nutrition standards to align with the Dietary Guidelines for Americans (DGA), there are no existing limitations or guidelines regarding sugar content of school meals [35]. The DGA recommend that sugar should account for less than 10% of total daily calories [36]. According to the American Academy of Pediatrics (AAP), all children should consume less than 6 teaspoons of added sugar per day, and younger children should consume even less with 3–8-year-olds being limited to 3 teaspoons of sugar per day [35]. While a complete nutrient analysis was beyond the scope of this initial project, the photographs did demonstrate that many of the school meals served during our study period were not meeting this daily sugar recommendation. According to the photographed nutrition labels, one pop-tart pastry, a common item provided in school breakfasts in the study, contains 15 g or nearly 4 teaspoons of added sugar—60% of the AAP’s daily recommendation. One previous study conducted before the pandemic found that 92% of U.S. schools exceeded the DGA for sugar in breakfast, and 69% of schools exceeded it for lunches [37]. A report published in November 2021 states that most companies supplying school food had about 75% compliance to DGA for added sugars in their foods [38]. However, parents in our study school districts perceived that school meals contain too much sugar and are unhealthy. Similarly, in a study of elementary school parents in Oregon, improving nutrition standards for school meals was noted as the top priority for supporting children’s healthy eating [39], so this concern is not unique to our study population. School districts must seize the opportunity to model healthy habits by providing meals that are healthy and in accordance with nutritional guidelines. Parent perceptions in this study strongly support the inclusion of a standard for added sugars in Child Nutrition Reauthorization, which is expected to be considered by Congress in the coming year. 

Our findings also indicated that meal appeal and quality were major concerns of parents. While some concerns raised in focus group discussions were specific to the pandemic constraints (e.g., need for individual packaging, transportation of meals from school to home), other concerns apply to school meals served during more routine school periods. Parents said that a lack of variety in food led to children refusing to eat the meals, which was remedied at home by introducing additional fresh ingredients that improved taste, presentation, and nutrition. For example, one parent added tomatoes, lettuce, and peppers to sandwiches provided by the school. In the New York City study cited earlier, parents expressed similar concerns to those identified in our study about meal appeal, noting repetitive and limited food options and unappealing presentation and texture of foods [27]. Similarly, in a prior study of parents of students in rural Midwestern middle schools, parents stated that the top reason why their child did not participate in school meals was because they did not like what was being served [40]. In contrast to other studies that describe advantages to serving pre-sliced fruit [41], parents in our study stated that their children desired more whole fresh produce as packaged, pre-sliced fruits and vegetables were often slimy, moldy, or otherwise unappealing. One innovative way to increase the variety of healthy, fresh foods is to source locally grown produce rather than mass-produced, packaged sliced fruits and vegetables. Research demonstrates that student and teacher school meal participation increased from 1.3 percent to 16 percent in school districts that implemented farm to school programs that increased local produce in the school meals [42]. 

Prior studies demonstrate that parents’ perception of school meals directly affects their child’s meal participation [43]. This was also reflected in our findings, as in discussion of wasted food, multiple parents stated they had either already stopped or planned to stop picking up school meals. Limited research suggests that parents may not know that meals must meet federal nutrition standards; they misperceive home meals as healthier than school meals, have concerns about the appeal and taste of food, or do not trust school meal quality [18,34,40,44]. While these findings are useful, generalizability is limited, as most studies do not capture rural areas like SJV, which face unique challenges. Though one in four US children are Latino, few studies explore such perspectives, and those that do note concerns about the cultural mismatch between Western foods offered at school and more traditional foods at home [45]. Our study adds the unique perspective of rural, mostly Spanish-speaking, Latino parents to the existing literature about parent perceptions of school meals, which is critically important to understand given this population’s high risk of food insecurity.

Given the unprecedented policy window with California’s new universal school meals policy and its capacity to reduce persistently high rates of FI and obesity stemming from the pandemic [34], the community-academic-policy team has worked to develop policy briefs based on study findings that recommend (1) including guidelines limiting added sugars in school meals in the upcoming Child Nutrition Reauthorization and (2) supporting programs that incentivize use of local fresh produce in school meals. The briefs were circulated among community members and nutrition policy advocates to support these efforts [46]. Additionally, our research and community partners are working to present photographs and findings to school districts and other stakeholders to advocate for improvements in school meals locally.

Limitations of our study include the fact that, due to safety constraints during the COVID-19 pandemic, focus group discussions were conducted virtually and required access to phone or computer and Wi-Fi. While this had the potential to bias our sample towards those with internet access, our community partners worked to help those with limited technology skills participate. The sample size of some of the small group interviews, which were as small as two participants, is a limitation for traditional focus group type discussions. Despite this, our facilitators encouraged highly interactive discussions of the designated photos. We believe that, in some cases, the smaller group discussions further accommodated language of choice and the ability to organize groups by school district. This facilitated presentation of focus group findings at the district level, which was important for advocacy at the local-district level in keeping with the priorities of photovoice methodology [28]. When reviewed separately from aggregate data, themes from the two to three person small group interviews were consistent with overall findings, and all major themes discussed in the group were also discussed in other larger focus groups. Lastly due to pandemic USDA school meal program waivers that facilitated meal distribution in different formats (e.g., grab-and-go) and provided more flexibility around nutrition standards, parents’ perceptions of foods as being unhealthy may not be representative of typical school meals served in a non-pandemic time period. However, these findings will be important to consider for future school closures due to COVID-19 surges, as well as in response to other disasters such as wildfires and hurricanes or during winter, spring, and summer instructional breaks.

## Figures and Tables

**Table 1:** Theme 1: Parent Descriptions of the Benefits of School Meal Distribution during COVID-19 Pandemic School Closures.

Description	Quotes	Photos
a. Convenience of pick-up and communication	“I like the locations because the schools are so close to where I live.” “I like that they call us for updates that makes me remember.”	
b. Financial benefit from school meals	“A positive aspect is knowing that we have food, that, if in this pandemic we really run out of food, there is milk for example. If it is a great help and I have four children, it is good to know that there is food for them there.” “Well for me in particular a benefit right now as things are, which will lower employment, it is helping me financially.”	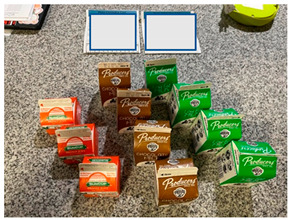 Bulk foods that support families financially by decreasing grocery needs for: (i) cartons of milk 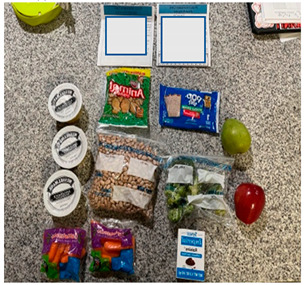 (ii) bulk staple items like a bag of dried beans
c. Pandemic safety	“Yes, I can easily access it and I enjoy that the staff are being safe for COVID-19 by wearing masks and things like that.”	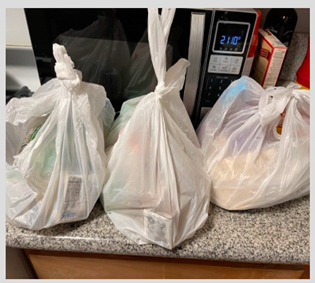 Meals in plastic bags that were perceived as improving pandemic safety.

**Table 2:** Theme 2: Parents’ Perceptions of the Quality and Appeal of School Meals during the COVID-19 Pandemic.

Description	Quotes	Photos
a. Wasted food due to unappealing meals	“They tell me they want to eat, but not the food here because it is bad quality. The meat has no flavor, and I even have to use the napkin to remove grease from the chicken nuggets.” “I went several times, and the fruit was bruised. I did not want them to eat it, so we threw it away and I would prefer not to throw away things.” “There is too much waste, so I decided not to go.”	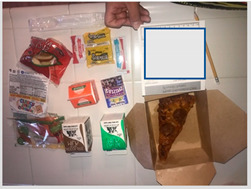 (i) Slice of pizza sitting in a pool of grease was unappealing to kids 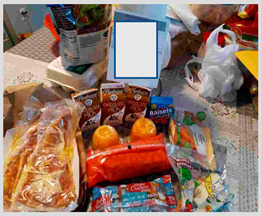 (ii) Shrink wrapped pizza slice that is squished and misshaped.
b. Lack of variety in meals	“I would like more options and less repetition. Then the kids get very tired and they just don’t eat it.”	
c. Meals required additional preparation prior to consumption	“You cannot eat it right away. You need to fix it to be edible to eat it.” “I will prepare it for my son. But I add the lettuce, I add the tomato, the onion so that he can eat it. But they don’t give it that way at school. The school gives it to him plain.”	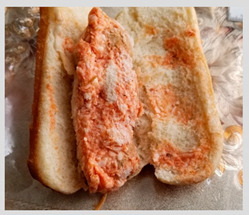 A sandwich with filling that was frozen
d. Flimsy packaging led to damaged food	“The bags for me always break especially in the handle part and I feel bad because the food might be damaged.”	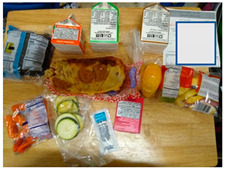 “Squished” pizza roll

**Table 3:** Theme 3: Parents’ Perceptions of the Healthfulness of School Meals Provided during the COVID-19 Pandemic.

Description	Quotes	Photos
a. Kids prefer fresh foods	“One good thing is that they had fresh food, but when they come as packaged, they kind of spoil faster because they are sealed up.” “They [children] say they want the whole fruit, banana, apple, and they don’t want it packaged.”	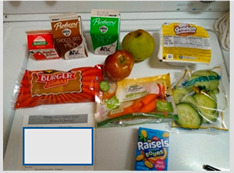 (i) Whole apple and pear were preferred to packaged and sliced cucumbers and carrots, which were perceived as slimy.
b. Food was perceived as too sweet and too greasy	“The cereal is too sweet, everything is not healthy in this bunch of food.” “I think the only thing there that I don’t like being given so much is Pop-tarts, because it has a lot of sugar” “It’s [school meals] crap crap food. It’s junk. Why are you going to put chips and fritos in front of the kids?” “Children cannot sustain themselves on treats that give pure sugar. They [schools] give for the morning bars and cereal that are full of sugar.”	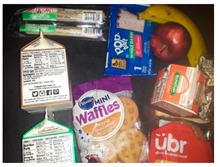 (i) Waffles and Pop-tarts were perceived as too sugary by parents. 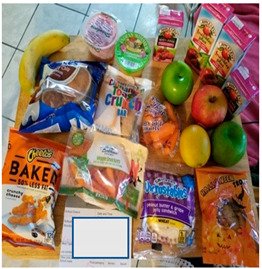 (ii) Chips (Cheetos) and sugary cereal (Cinnamon Toast Crunch) were offered in the meals.
c. Milk as a healthy staple food	“I like the milk because it helps lessen how much I have to buy from the store in addition to the fruit. This is helpful to me.” “I also like the milk because it lowers the cost of my monthly grocery bill. It can be used for cereal and things like that”.	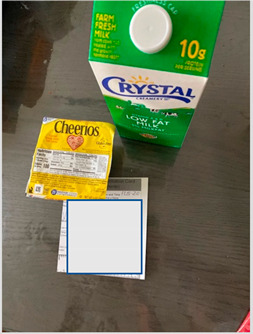 ½ gallon milk carton described as helping lower grocery bill.
d. School meals set a poor example for a healthy lifestyle	“I think there needs to be more education and healthier choices… The school needs to be responsible for showing meals that are healthy, but also that the kids would actually enjoy. That is my point of view.” “My child commented to me, how do you expect me to eat healthy and good if I only like the milk out of the lunches we get? That is concerning to me because I want them [children] to be able to get the nutrients they need to do well in school and be healthy.” “If the district wants children to eat healthy, but they are giving fatty food, they are giving a lot of cheese and it has a lot of fat, cholesterol rises and that children are consuming this at an early age also affects them.”	* 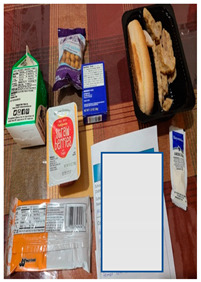 * The food above was described as including an unhealthy breakfast of a “high-calorie bar” and lunch of bread with no vegetables.

## Data Availability

The data presented in this study are available on request from the corresponding author. The data are not publicly available due to protections for privacy of community members and participants as this is a qualitative study and identifiers are present throughout the transcripts.
